# Eliater: a Python package for estimating outcomes of perturbations in biomolecular networks

**DOI:** 10.1093/bioinformatics/btae527

**Published:** 2024-08-26

**Authors:** Sara Mohammad-Taheri, Pruthvi Prakash Navada, Charles Tapley Hoyt, Jeremy Zucker, Karen Sachs, Benjamin M Gyori, Olga Vitek

**Affiliations:** Barnett Institute for Chemical and Biological Analysis, Northeastern University, Boston, MA, 02115, United States; Khoury College of Computer Sciences, Northeastern University, Boston, MA, 02115, United States; Khoury College of Computer Sciences, Northeastern University, Boston, MA, 02115, United States; Khoury College of Computer Sciences, Northeastern University, Boston, MA, 02115, United States; Pacific Northwest National Laboratory, Richland, WA, 99354, United States; Next Generation Analytics, Palo Alto, CA, United States; Modulo Bio, Inc., Los Altos, CA, 92121, United States; Barnett Institute for Chemical and Biological Analysis, Northeastern University, Boston, MA, 02115, United States; Khoury College of Computer Sciences, Northeastern University, Boston, MA, 02115, United States; Department of Bioengineering, Northeastern University, Boston, MA, 02115, United States; Barnett Institute for Chemical and Biological Analysis, Northeastern University, Boston, MA, 02115, United States; Khoury College of Computer Sciences, Northeastern University, Boston, MA, 02115, United States

## Abstract

**Summary:**

We introduce Eliater, a Python package for estimating the effect of perturbation of an upstream molecule on a downstream molecule in a biomolecular network. The estimation takes as input a biomolecular network, observational biomolecular data, and a perturbation of interest, and outputs an estimated quantitative effect of the perturbation. We showcase the functionalities of Eliater in a case study of *Escherichia coli* transcriptional regulatory network.

**Availability and implementation:**

The code, the documentation, and several case studies are available open source at https://github.com/y0-causal-inference/eliater.

## 1 Introduction

Estimating the effect of perturbations of biomolecules (such as silencing a gene or altering the function of a protein with a drug) on their descendants is a fundamental task in the analysis of biomolecular networks. It aids in uncovering causal relationships within complex biological systems, and informs drug discovery and personalized medicine efforts.

The causal effect of an intervention is typically assessed experimentally, where biological replicates are subjected to a perturbation, and compared to controls. The difference in the outcome between the two groups determines the causal effect. However, perturbations are often impractical to implement, or are unethical. Therefore, of interest are model-based methods of causal inference (Pearl 2009, Bhattacharya *et al.* 2022) that estimate causal effects from observational experiments, i.e. experiments observing the system without the perturbation, in its natural state.

Model-based causal inference methods require three inputs. The first is a network structure in the form of a directed acyclic graph (DAG), extracted from literature and available in databases such as INDRA (Bachman *et al.* 2023). The second input is an observational experiment quantifying the state or abundance of biomolecules represented in the network, e.g. using transcriptomic or proteomic technologies. The third input is a causal query of interest, i.e. the causal effect of perturbing an upstream biomolecule *X* (exposure) on downstream biomolecule *Y* (outcome). The output is a probability distribution of the causal effect, or its expected value.

Model-based causal inference methods are flexible. They can accommodate a diversity of assumptions regarding the data (Bhattacharya *et al.* 2022), as well as latent confounders (i.e. unmeasured variables that affect both the target of intervention and the outcome) (Pearl 2009, Mohammad-Taheri *et al.* 2022). However, they currently fall short in biomolecular applications, for several reasons. First, while biomolecular network models can include hundreds of variables, many of these may not be necessary for the causal query estimation (Mohammad-Taheri *et al.* 2023). In fact, incorporating all observable variables into query estimation can lead to bias or increase asymptotic variance (Shpitser and Pearl 2008, Cinelli *et al.* 2022). Second, since biological systems are dynamic and context-dependent, the context and the resolution of the network and of the data may mismatch, leading to bias in causal query estimation. Finally, while some functionalities are accessible through open-source packages in R (Scutari 2010, Tikka and Karvanen 2017) or Python (Ankan and Panda 2015, Hoyt *et al.* 2021, Bhattacharya *et al.* 2022, Blöbaum *et al.* 2022), others, such as detecting and removing nuisance variables, are not. Users must have substantial experience with the available resources, be able to integrate them across diverse programming languages and data structures, and directly implement the parts that lack implementations.

To overcome these limitations, this manuscript introduces Eliater, a Python package implementing current best practices in causal inference, specifically tailored for supporting causal query estimation in biomolecular networks. Eliater checks the mutual consistency of the network structure and observational data with conditional independence tests, checks if the query is estimable from the available observational data, detects and removes nuisance variables unnecessary for causal query estimation, generates a simpler network, and identifies the most efficient estimator of the causal query. Eliater returns an estimated quantitative effect of the perturbation.

## 2 Background

**Figure 1.**
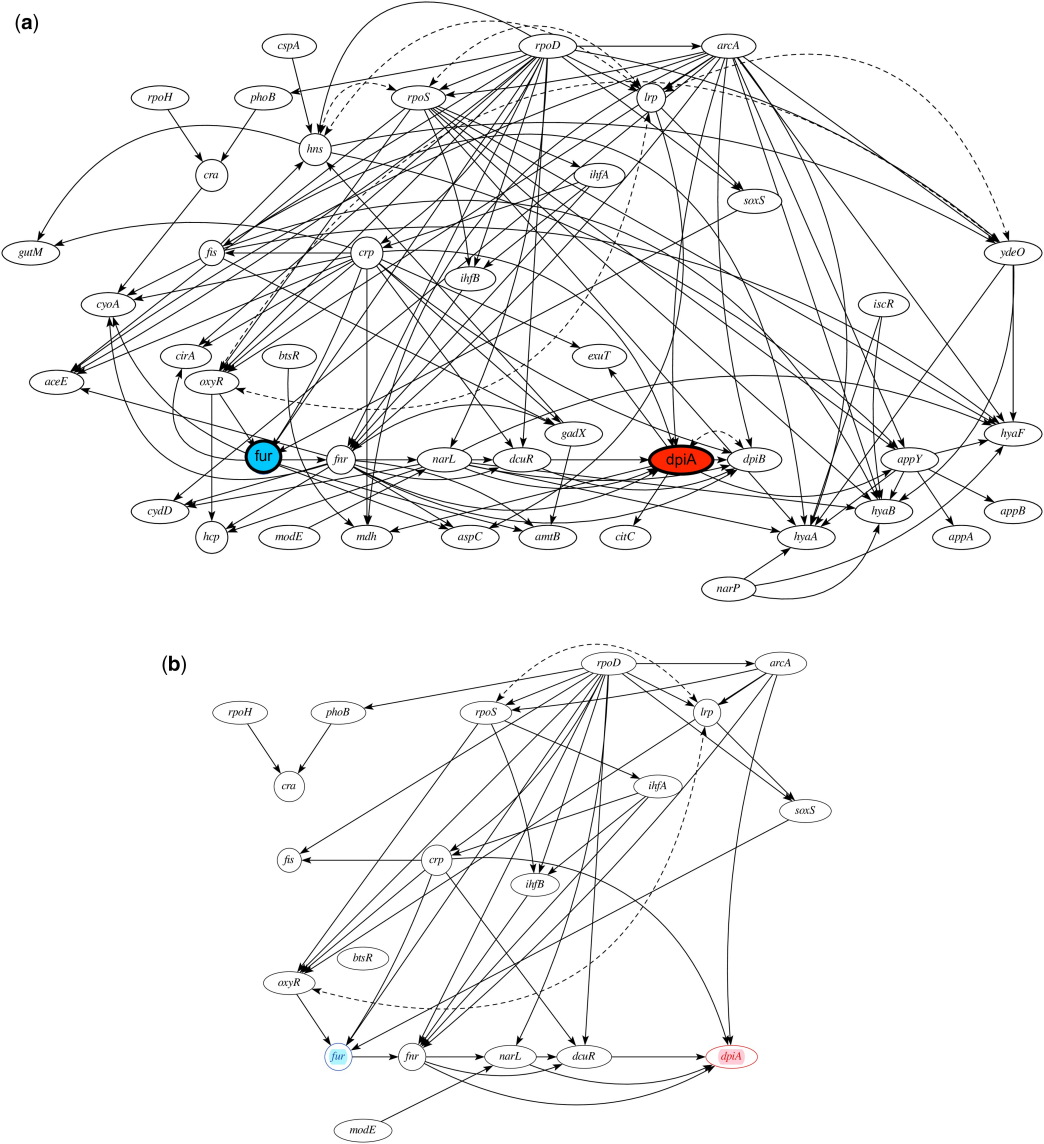
Case study 3. *Escherichia coli* K-12 transcriptional network. (a) The original network with 44 observed and 7 latent nodes, connected by a total of 122 directed, and 7 undirected edges. (b) The simplified network, with all the nuisance variables removed. It contains 20 nodes, and 38 directed and 2 undirected edges. Fur gene is the exposure and dpiA gene is the outcome.

**Causal models of biomolecular networks** In causal inference applications, the models are represented as DAGs. When a DAG includes unmeasured latent variables, it can be efficiently represented by a DAG that only includes exogenous latent variables (i.e. variables without any parents). This is accomplished by four simplification rules in Evans (2016) that preserve the joint probability distribution over the observable variables. The simplified DAG is then converted to an acyclic directed mixed graph (ADMG) (Richardson *et al.* 2023) by replacing the exogenous latent variables with bi-directed edges as in Fig. 1a. The ADMG structure allows us to misspecify the number of latent variables without undermining the precision of causal query estimation, as long as the graph accurately specifies the structure of the observable variables (Mohammad-Taheri *et al.* 2022).

**Causal query** A causal query *Q_G_* with respect to a DAG or an ADMG *G* is the effect of an intervention on a set of treatment or exposure variables **X** on a set of observable variables **Y**. The intervention forces **X** to take fixed values irrespective of their natural causes, and is denoted do(X=x). *Q_G_* takes probabilistic form such as P[Y|do(X=x)].

**Causal query identification** takes as input a DAG or an ADMG *G*, and a set of observable variables, and examines whether the causal query *Q_G_* is estimable without bias (Pearl 2009). The identification can be achieved by the ID algorithm (Shpitser and Pearl 2006).

**Mutual consistency of graph and data** A network in the form of a DAG or ADMG implies conditional independencies of its variables. We can assess whether these conditional dependencies are consistent with the conditional independencies in observational data using frequentist tests such as $\chi^{2}$ test for categorical variables, or the Pearson test for Gaussian data (Scutari 2010). Rejecting the null hypothesis implied by the DAG indicates mutual inconsistencies.

**Causal effect estimators** Several Python and R packages implement estimators of causal effects. For example Python packages ananke (Bhattacharya *et al.* 2022) and DoWhy (Blöbaum *et al.* 2022) offer non-parametric or semi-parametric estimators that are asymptotically unbiased, computationally efficient, and free from parametric assumptions. If the network variables are linearly related, these packages offer parametric linear regression restimator. However, the estimators are restricted to queries in the form of average treatment effect (ATE) E[Y|do(X=1)]−E[Y|do(X=0)], and do not eliminate the nuisance variables. In addition, these estimators are designed for general use, and do not address issues specific to biomolecular networks, such as the possibility of mutual inconsistency between the network structure and data, and the need for simplifying graph structures to remove unnecessary variables and improve the interpretability of the results. This manuscript contributes a comprehensive end-to-end analytical Python package for causal query estimation that addresses these issues.

## 3 Description

### 3.1 Input and output

Eliater requires three main inputs.

**Biomolecular network** The first input is a biomolecular network of any type (such as regulatory, signaling or metabolic, with both directed and undirected edges), extracted from existing knowledge bases such as INDRA (Bachman *et al.* 2023). The network is specified in the form of DAGs or ADMGs. Eliater has been tested on networks with up to 44 nodes and 129 edges.

**Observational data** The second input to the workflow is an observational dataset that quantifies the abundance of biomolecules at steady state across multiple biological replicates. The biological replicates must have natural variation, which can be achieved by lightly stimulating the system. The data can be continuous or discrete, acquired with a targeted acquisition technology such as antibody-based assays, or broad-coverage discovery technology such as transcriptomics or proteomics. Importantly, the nature of the data has to match the nature of the network. For example, if the experimental measurements only quantify protein abundance, the network has to reflect causal events that affect the abundance (as opposed to other events such as post-translational modifications). Eliater represents observational data as pandas (The Pandas Development Team 2024) data frames. It has been tested in experiments with at least 20 biological replicates.

**Causal query** The final input is a causal query, i.e. the causal effect of the biomolecule *X* that is the target (or exposure) of the intervention, on the biomolecule *Y* that is the outcome of interest. Eliater supports queries in the form of ATE, expected value E[Y|do(X=x)], or probability distribution P[Y|do(X=x)] with a single binary or continuous exposure, and single outcome.

**Output** Eliater estimates the outcome of the perturbation in form of a probability distribution, or its expected value.

### 3.2 Functionalities of Eliater

Eliater proceeds in five steps.

**Step 1: Verify mutual consistency of graph and data** Eliater finds all the conditional independencies implied by the graph, performs conditional independence tests, and reports the percentage of failures. A large number of failed tests indicates that the network and the data are not mutually consistent, and the estimate of the causal effect may be biased. Eliater addresses this by repairing the network and introducing bi-directed edges between variables with failed tests. For example, if the conditional independence of *X* and *Y* given *Z* fails, this indicates that there are likely some unobserved common causes (confounders) of both *X* and *Y*. Alternatively, users may opt to revise the network structure or the dataset.

**Step 2: Check query identifiability** Given a mutually consistent network and data, Eliater checks if the query is estimable from the observational data by the ID algorithm. If the query is not identifiable, additional experiments may be required to augment the data and make the query identifiable.

**Step 3: Find nuisance variables** Not all the network variables are required for causal query estimation. In fact, including some of the variables in causal query estimation may inflate both bias and asymptotic variance of the estimator. Following (Shpitser and Pearl 2008, Cinelli *et al.* 2022), Eliater detects these variables and marks them as latent. It is worth noting that detecting and marking nuisance variables as latent may have a small effect on the precision of causal query estimation. However, it helps focus on necessary variables for causal query estimation without losing precision (Cinelli *et al.* 2022). We are not aware of an alternative implementation of this step in other packages.

**Step 4: Simplify the network** Eliater takes as input the output network from Step 3, applies the simplification rules in Evans (2016), and produces a new network where the nuisance variables are removed. Since the joint probability distribution of observable variables in the simplified network is equivalent to that in the input network, the step does not impact the accuracy of query estimation. However, the simplification reduces a large problem to a smaller and more tractable one.

**Step 5: Estimate the causal query** For systems with non-Gaussian measurements and non-linear relationships between variables, Eliater supports the non-parametric and semi-parametric estimators from the ananke package, and automatically selects the most efficient estimator. Currently, Eliater only supports queries in the form of ATE with single binary exposure and single outcome.

For systems with approximately Gaussian measurements and linear relationships, Eliater implements a linear regression estimator. Currently, it supports single discrete or continuous exposure and single continuous outcome. It supports estimators of more query types, including ATE, point estimators of the expected value E[Y|do(X=x)], and estimators of probability distribution P[Y|do(X=x)]. The latter is used to derive interval estimators that characterize estimation uncertainty.

### 3.3 Implementation and documentation

Eliater requires Python version 3.11 or later. Its dependencies include y0 (Hoyt *et al.* 2021), ananke (Bhattacharya *et al.* 2022), and pgmpy (Ankan and Panda 2015). The implementation uses a consistent set of data structures throughout the workflow, and its modular design facilitates future extensions. For user convenience, Eliater specifies numerous default parameter settings, while providing the flexibility for input parameters controls. Eliater documentation includes four case studies representing diverse topologies of biomolecular networks, observational data types and causal query types. The case studies took between 5 and 30 min on a macOS Monterey system with 1.6 GHz Dual-Core Intel Core i5.

## 4 Case study of *Escherichia coli* K-12 transcriptional network

Below, we provide details of one of the case studies in Eliater documentation. It demonstrates the effect of detecting and removing nuisance variables, and transforming a relatively complex biomolecular network into a smaller network with a minimal set of variables needed for causal query estimation.

**Input 1: Biomolecular network** A transcriptional regulatory network motif of *E.coli* in Fig. 1a modeled the molecular interactions within a bacterial cell. The network was manually extracted from the EcoCyc database (Keseler *et al.* 2021).

**Input 2: Causal query** The query of interest was the expected expression of gene *dpiA* upon setting the expression of gene *fur* to 0, i.e. $Q_{\mathrm{fur}}=E[\mathrm{dpiA}|do(\mathrm{fur}=0)]$. This is a representative example of queries that can be used to estimate the effect of drugs or perturbations. Estimation of this query is challenged by the presence of several confounders.

**Input 3: Observational data** The observational data consisted of 260 RNA-seq expression profiles of *E.coli* K-12 MG1655 and BW25113 strains, spanning 154 distinct experimental conditions. The continuous normalized expression values were retrieved from the PRECISE database (Sastry *et al.* 2019), https://github.com/SBRG/precise-db.

**Evaluation criterion** The PRECISE database included an interventional experiment, where the expression of *fur* was inhibited and set to zero. The interventional experiment had three biological replicates. Therefore, the ground truth for validation is the mean expression of *dpiA* over the three interventional replicates, namely 4.9.

**Step 1: Verify mutual consistency of graph and data** Eliater assessed the mutual consistency of the network structure and of the continuous observational data using the Pearson test with a significance level of 0.01. Since the number of all possible conditional independence tests implied by this network was over a million, we set the maximum number of variables to condition upon to three. This implied 498 tests, 194 (39%) of which failed. This is likely due to the fact that the observational data involved 154 conditions, and the single network structure only partially matched the transcriptional regulation events under all these conditions.

We performed two analyses. In the first, we repaired the network structure according to Step 1 of Eliater. The analysis added a total of 106 bi-directed edges to the original network in Fig. 1a. The resulted network can be found in the notebook section of Eliater. In the second analysis, we proceeded with the original network without repair.

**Step 2: Check query identifiability** In both analyses the ID algorithm found the query identifiable, and we proceeded to Step 3.

**Steps 3 and 4: Find nuisance variables and simplify the network** Given the query and the data, Eliater identified a total of 21 nuisance variables, marked them as latent, and simplified the network. For the analysis without the repair, the simplified network is shown in Fig. 1b. This network is smaller, and therefore easier to visualize and interpret. For the analysis with network repair, the simplified network was the same as in Fig. 1b, but with 106 additional bi-directed edges. It can be found in the notebook section of Eliater.

**Step 5: Estimate the causal query** Since the case study had a non-binary exposure, continuous outcome, and the variables could be thought of as approximately linearly associated, we used the linear regression estimator from Eliater.

**Estimated causal query** The estimated causal query $\hat{E}[\mathrm{dpiA}|do(\mathrm{fur}=0)]$ for the repaired network was 4.4 and for the non-repaired network was 4.07. This indicated that the inhibition of gene *fur* leads to an increase in the activity of gene *dpiA*. The estimated value in both cases was of the same sign, and within 10% difference from the ground truth of 4.9.

## 5 Discussion

We introduced Eliater, an open-source Python package for estimating the effect of perturbation of an upstream biomolecule on a downstream biomolecule. Eliater is compatible with a variety of biomolecular networks, with observational data acquired with a variety of experimental technologies, and with a variety of causal query types.

The proposed workflow has several limitations. First, it is restricted to capturing the steady-state behavior of a biological system. The acyclic nature of DAG and ADMG input graphs cannot explicitly capture feedback effects, or other temporal patterns that has non-trivial implications for causal query estimation. Second, the approach relies on frequentist tests of conditional independence, which have well-known drawbacks such as the dependencies of the *P*-values on the sample size, challenges of scalability, and multiple testing. Third, Eliater currently addresses the repair of network structure by adding bi-directional edges between variables with failed tests. This may introduce false positive edges, and render the query unidentifiable. Future research directions include transitioning away from frequentist tests, incorporating modern causal structure learning algorithms to more effectively repair the networks, and developing functionalities for working with a broader family of graphs. Despite these opportunities for improvements, the case studies in the package documentation demonstrate the utility of Eliater in its current form in a range of practical applications.
